# Rapid HER2 cytologic fluorescence in situ hybridization for breast cancer using noncontact alternating current electric field mixing

**DOI:** 10.1002/cam4.3626

**Published:** 2020-12-06

**Authors:** Shin‐nosuke Watanabe, Kazuhiro Imai, Hiroshi Nanjo, Yuki Wakamatsu, Yoshihiko Kimura, Yoshihisa Katayose, Shuichi Kamata, Kaori Terata, Eriko Takahashi, Ayano Ibonai, Ayuko Yamaguchi, Hikari Konno, Misako Yatsuyanagi, Chiaki Kudo, Shinogu Takashima, Yoichi Akagami, Ryuta Nakamura, Yusuke Sato, Satoru Motoyama, Kyoko Nomura, Yoshihiro Minamiya

**Affiliations:** ^1^ Department of Thoracic Surgery Akita University Graduate School of Medicine Akita Japan; ^2^ Department of Pathology Akita University Graduate School of Medicine Akita Japan; ^3^ Akita Kousei Medical Center Akita Japan; ^4^ Akita City Hospital Akita Japan; ^5^ Japanese Red Cross Akita Hospital Akita Japan; ^6^ Akita Industrial Technology Center Akita Japan; ^7^ Department of Environmental Health Science and Public Health Akita University Graduate School of Medicine Akita Japan

**Keywords:** breast cancer, cytology, human epidermal growth factor receptor 2, in situ hybridization, noncontact alternating current electric field mixing

## Abstract

**Background:**

Human epidermal growth factor receptor 2‐in situ hybridization (HER2‐ISH) is widely approved for diagnostic, prognostic biomarker testing of formalin‐fixed paraffin‐embedded tissue blocks. However, cytologic ISH analysis has a potential advantage in tumor samples such as pleural effusion and ascites that are difficult to obtain the histological specimens. Our aim was to evaluate the clinical reliability of a novel rapid cytologic HER2 fluorescence ISH protocol (rapid‐CytoFISH).

**Materials and Methods:**

Using a new device, we applied a high‐voltage/frequency, noncontact alternating current electric field to tissue imprints and needle rinses, which mixed the probe within microdroplets as the voltage was switched on and off (AC mixing). Cytologic samples (n = 143) were collected from patients with immunohistochemically identified HER2 breast cancers. The specimens were then tested using standard dual‐color ISH using formalin‐fixed paraffin‐embedded tissue (FFPE‐tissue DISH) for HER2‐targeted therapies, CytoFISH, and rapid‐CytoFISH (completed within 4 h).

**Results:**

All 143 collected cytologic specimens (50 imprinted cytology specimens from resected tumors and 93 liquid‐based cytology specimens from needle rinses) were suitable for FISH analysis. The HER2/chromosome enumeration probe (CEP) 17 ratios did not significantly differ between FFPE‐tissue DISH and either CytoFISH protocol. Based on HER2 scoring criteria, we found 95.1% agreement between FFPE‐tissue DISH and CytoFISH (Cohen's kappa coefficient = 0.771 and 95% confidence interval (CI): 0.614–0.927).

**Conclusion:**

CytoFISH could potentially serve as a clinical tool for prompt determination of HER2 status in breast cancer cytology. Rapid‐CytoFISH with AC mixing will enable cancer diagnoses and HER2 status to be determined on the same day a patient comes to a clinic or hospital.

## Graphical abstract

Rapid in situ hybridization device used to apply an alternating current electric field analysis of cytologic specimens (Rapid CytoFISH) has a potential advantage in tumor samples such as pleural effusion and ascites that are difficult to obtain the histological specimens. Rapid‐CytoFISH will enable cancer diagnoses and Human epidermal growth factor receptor 2 (HER2) status to be determined on the same day a patient comes to a clinic or hospital.

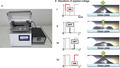

## 1 INTRODUCTION

Human epidermal growth factor receptor 2 (HER2) is an important prognostic and predictive biomarker in breast cancer.^1^, ^2^ Amplification of HER2 is a primary driver mutation that occurs in approximately 20% of breast cancers and is associated with a poor prognosis in untreated patients.^2^, ^3^ Because HER2‐targeted agents are an effective therapeutic approach for patients with overexpressed HER2 advanced/metastatic breast cancer,^2^, ^3^, ^4^, ^5^, ^6^, ^7^ HER2 levels should be assessed in all breast cancers. The commonly used techniques to determine HER2 status are immunohistochemistry (IHC) and in situ hybridization (ISH).

HER2‐ISH is a validated, reproducible method that is widely approved for diagnostic, prognostic biomarker testing of formalin‐fixed, paraffin‐embedded tissue blocks (FFPE).^8^ However, it is associated with several limitations: 1) the FFPE procedure involves the use of toxic formalin; 2) the fixation and paraffin embedding process can be time consuming; and 3) the subsequent probe hybridization takes an additional 14–18 hours. As a result, it may take several days to assess HER2 status when using the conventional ISH procedure with FFPE. Moreover, because nearly all hospitals outsource performance of these assays, time‐consuming ISH can delay the treatment intervention for patients with advanced breast cancer for as long as several weeks. In contrast, cytology enables same‐day diagnosis, which decreases patient anxiety and facilitates treatment planning. In addition, cytological techniques, including flow cytometric analysis of DNA ploidy, immunocytology, and ISH for cancer detection, are being increasingly applied to facilitate the identification of neoplastic cells for molecular diagnoses.^9^ In breast cancer, for example, use of cytologic specimens for fluorescence ISH (FISH) to evaluate HER2 status revealed that FISH analysis of cytologic specimens produced more accurate HER2/chromosome enumeration probe (CEP) 17 signal ratios and HER2 copy numbers than histological specimens, as CEP17 is easily lost during histological sectioning.^9^, ^10^, ^11^, ^12^, ^13^, ^14^, ^15^ Another potential advantage of cytologic FISH is that it is difficult to obtain histological tumor specimens from some metastatic sites, such as plural effusion and cerebrospinal fluid. Moreover, a rapid 1‐day HER2‐ISH protocol for breast cancer cytology is not yet available to diagnosticians/surgeons.

We have been developing a rapid‐IHC/ISH that makes use of an alternating current (AC) electric field to facilitate the reagent reaction, and have reported its usefulness for molecular target detection in lung cancer, breast cancer, and brain tumors.^16^, ^17^, ^18^, ^19^ The device reduces the time required for IHC/ISH as well as the amount of reagent required for these analyses. This device applies a high‐voltage, low‐frequency, or high‐frequency AC electric field to the sections. The resultant coulomb force stirs the diluted solution within microdroplets on the sections because the voltage is switched on and off at specific intervals. It increases the opportunity for antibody–antigen or DNA probe‐gene contact (AC mixing). This rapid‐IHC method enables rapid detection of target cells in frozen sections and cytological samples, and can provide us with an intraoperative diagnosis within 20 minutes.^16^, ^17^ As a further application, we demonstrated that rapid‐ISH with AC mixing can be used to detect HER2 amplification within 6 hours in breast cancer patients ^20^, ^21^ and can detect Anaplastic lymphoma kinase break‐apart hybridization within 4.5 hours in lung cancer patients.^22^, ^23^ Moreover, in our earlier HER2 rapid‐ISH study using FFPE, we obtained 98.8% agreement between amplification status detected with conventional ISH and our new rapid‐ISH.^20^ Although the rapid‐ISH with AC mixing still has limitations, we anticipate that this technique will be applicable in multiple settings.

Cytologic specimens also have a potential advantage for evaluating HER2 status, especially in tumor samples such as serous effusion in the body cavity that are difficult to obtain FFPE tissue. Moreover, cytology can be useful because same‐day diagnosis decreases patient anxiety and facilitates prompt treatment, while the ISH procedure with FFPE in our earlier HER2 rapid‐ISH study is time consuming by the paraffin embedding process. In the present study, we evaluated the clinical utility and reliability of a novel rapid cytologic HER2 FISH protocol within 1 day (rapid‐CytoFISH).

## 2 MATERIALS AND METHODS

### 2.1 Patients

All experimental protocols were approved by the institutional review board at Akita University Hospital (Permit number: 1282 and 1408), and written informed consent was obtained from all patients. Breast cancer samples were collected through core needle biopsy (CNB) and/or surgery before deciding final pathologic diagnosis at the Akita University Hospital, Akita Kousei Medical Center, Akita City Hospital, or Akita Red Cross Hospital between October 2018 and March 2020. The patients’ clinical characteristics are listed in Tables 1 and 2. Used in this study were 50 imprinted cytology samples from surgical specimens and 93 liquid‐based cytology (LBC) samples that had been identified as HER2 0/(1+), (2+), or (3+) using IHC. In addition, the specimens were subjected to standard dual ISH using FFPE tissue (FFPE‐tissue DISH) using the ZytoVision manual protocol, or using an automated slide stainer. FFPE‐tissue DISH for HER2 was performed to determine whether to treat with HER2‐targeted therapies.

**TABLE 1:** Characteristics of patients from whom surgical cytology specimens were collected

Number of patients	50
Female sex, n (%)	100
Age	
Median	64.5
Range	38–89
Number of specimens	
Right	27
Left	23
Tumor size	
Tis	1
T1	29
T2	13
T3	2
T4	5
Lymph node	
Negative	37
Positive	13
Histology	
Ductal carcinoma in situ	1
Invasive ductal carcinoma	38
Invasive lobular carcinoma	6
Mucinous carcinoma	5
Others	0
Hormone Receptor Status	
ER	
Negative	3
Positive	47
PgR	
Negative	19
Positive	31
HER2‐IHC	
0	15
1+	14
2+	17
3+	4

**TABLE 2:** Characteristics of patients from whom liquid‐based cytology specimens were collected

Number of patients	93
Female sex, n (%)	98.90%
Age	
Median	68
Range	40–89
Number of specimens	
Right	47
Left	46
Tumor size	
Tis	4
T1	34
T2	36
T3	7
T4	10
Lymph node	
Negative	66
Positive	25
Metastasis	
Negative	86
Positive	5
Site*	
Lung	3
Liver	2
Bone	2
Histology	
Ductal carcinoma in situ	4
Invasive ductal carcinoma	78
Invasive lobular carcinoma	4
Mucinous carcinoma	5
Others	2
Hormone Receptor Status	
ER	
Negative	3
Positive	47
PgR	
Negative	19
Positive	31
HER2‐IHC	
0	15
1+	14
2+	17
3+	4

Abbreviations: ER, estrogen receptor; IHC, immunohistochemistry; PgR, progesterone receptor.

* Sites of metastasis were overlapping for patients.

### 2.2 Samples and preparation

Using standard histological techniques, CNB and surgical specimens were fixed in 10% formalin, embedded in paraffin, and stained using hematoxylin and eosin (HE) staining, IHC, and DISH. Imprinted cytology slides were obtained from resected breast cancer samples and were immediately fixed in 95% ethanol until they were used. LBC from CNB rinse samples were carried out using the concentration method with BD CytoRich systems Red (BD Franklin Lakes, NJ, USA), which involved rinsing the needle in a proprietary preservative solution. Both types of sample were re‐fixed in Carnoy's Solution for 15 minutes and 10% formalin for 5 minutes prior to FISH.

### 2.3 Immunohistochemistry

HER2‐IHC with a rabbit anti‐HER2/neu (4B5) monoclonal antibody (Roche Diagnostics, Penzberg, Germany) as the primary antibody was performed using a BenchMark XT Staining Platform (Ventana Medical Systems, Tucson, AZ) with an automatic staining protocol. HER2‐IHC were performed on all samples.

### 2.4 Dual‐color in situ hybridization (DISH)

Manual DISH was performed using a DNA‐specific dual‐color probes ZytoDot2C SPEC HER2/CEN 17 Probe Kit (ZytoVision, Bremerhaven, Germany), or INFORM HER2 Dual ISH DNA Probe Assays were performed on a BenchMark XT Staining Platform (Ventana Medical Systems, Tucson, AZ) according to the manufacturer's instructions.

### 2.5 New rapid cytological fluorescence in situ hybridization (rapid‐CytoFISH) using noncontact alternating current electric field mixing

**FIGURE 1:**
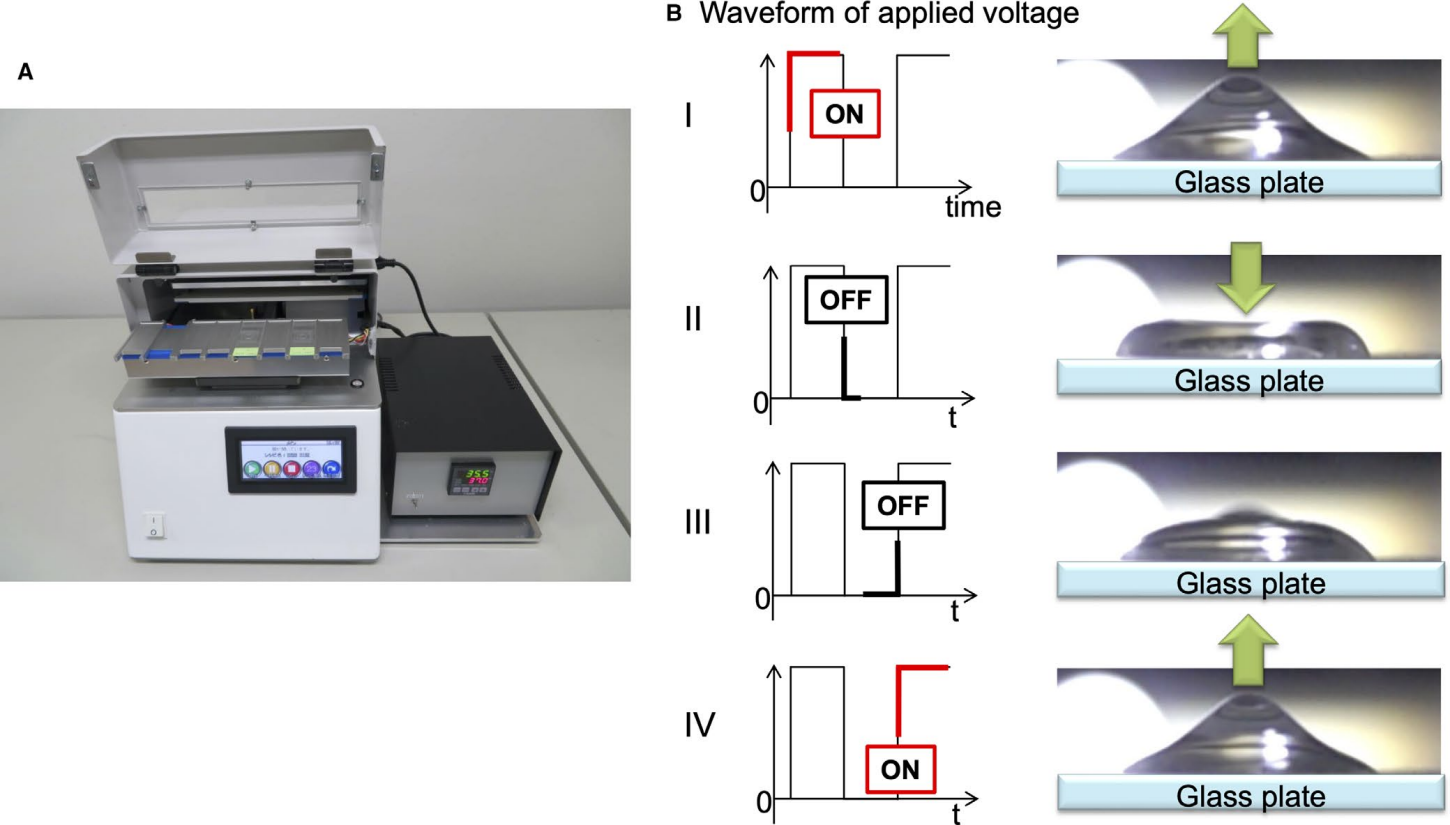
Rapid in situ hybridization device is used to apply a high‐voltage, high‐frequency alternating current electric field. (A) The prototype device mounting a temperature control unit. (B) Schema of the stir within a microdroplet as the voltage is switched on and off. The resultant coulomb force stirs the microdroplets on the sections, which increases the opportunity for DNA probe‐gene contact (AC mixing) because the voltage is switched on and off at specific intervals

After mounting a temperature control unit on our device, we used the prototype for rapid‐ISH, which we described in earlier reports.^20^, ^21^, ^22^, ^23^ The theory behind AC electric field mixing and the method for its application were described in detail previously (Figure 1).^16^, ^17^, ^18^, ^19^, ^20^, ^21^, ^22^, ^23^ Briefly, we use a device to apply an AC electric field to the sections. This causes the DNA probes in microdroplets to be mixed without a stirrer as the voltage is switched on and off at regular intervals.

HER2 CytoFISH was performed on cytological samples using a Histra‐HER2 FISH kit (In Vitro Diagnostics, JOKOH, Shizuoka, Japan). After mounting an insoluble label cover with a 1‐cm diameter hole in the center (SLS/E‐bar rabel II, Roche Diagnostics, Penzberg, Germany) on each microscope slide, 10 µl of HER2/neu rhodamine DNA probe and Ch‐17 fluorescein DNA probe (Histra‐HER2 FISH kit, In Vitro Diagnostics, JOKOH, Shizuoka, Japan) were applied evenly. Thereafter, 40 µl of HAIKORU‐K140N (KANEDA, Japan), which is a very low viscosity liquid paraffin, were added as an oil cover for preventing probe evaporation. The slide was then placed between the electrodes, and a high‐voltage (4.5 KV, offset 2.4 KV)/frequency (90 Hz) AC current was applied. There was a distance of 7.0 mm between the slide and electrode plates, and the current was applied for 180 minutes at 37°C (rapid‐CytoFISH protocol), as compared to 14–18 hours of hybridization in the standard CytoFISH protocol. Finally, the slides were counterstained with 10 µl of 4,6‐diamidino‐2‐phenylindole dihydrochloride (DAPI) and evaluated using fluorescence microscopy.

Table 3 summarizes each procedure required for ISH. The HER2 amplification based on the dual‐probe HER2/CEP17 ratio with an average HER2 copy number and signals/cell were evaluated using the ASCO/CAP scoring criteria including the updated 2018 guideline modifications^24^ with both the standard FISH and CytoFISH protocols.

**TABLE 3:** Procedural details for conventional FISH and CytoFISH

Protocol	Conventional	CytoFISH	Rapid‐CytoFISH
Dewaxing	27 min	none	none
		re‐fixed by Carnoy's 15 min	re‐fixed by Carnoy's 15 min
Activation, Dehydration, and Proteinase	40 min (37°C)	10 min, +10% formalin 5 min	10 min, +10% formalin 5 min
Denaturation and hybridization	14–18 h (37°C)	14–18 h (37°C)	180 min, AC mixing
Washing slides and other steps	8 min	8 min	8 min
Total time	20 h	18 h	**3 h 46 min**

Abbreviation: AC, alternating current electric field.

### 2.6 Statistical analysis

Statistical analysis was performed using JMP IN 14.2.0 software (SAS Institute, Cary, NC, USA). Differences among the groups were compared using the Dunnett's multiple comparison test. Cohen's kappa coefficient was used to assess the agreement of 4x2 contingency tables between protocols.

## 3 RESULTS

HER2 scoring using IHC and FFPE‐tissue DISH was performed with all specimens collected (50 imprinted cytology and 93 LBC samples) from 143 breast cancer patients. Using IHC, 49 specimens were scored 0, 49 were scored (1+), 31 were scored (2+), and 14 were scored (3+). The HER2 status of the specimens that were HER2 FFPE‐tissue DISH negative or positive were similarly evaluated using CytoFISH. The patient characteristics, including HER2/CEP17 ratios determined using CytoFISH, are listed in Tables 1 and 2. All of the cytologic specimens were suitable for FISH analysis.

**FIGURE 2:**
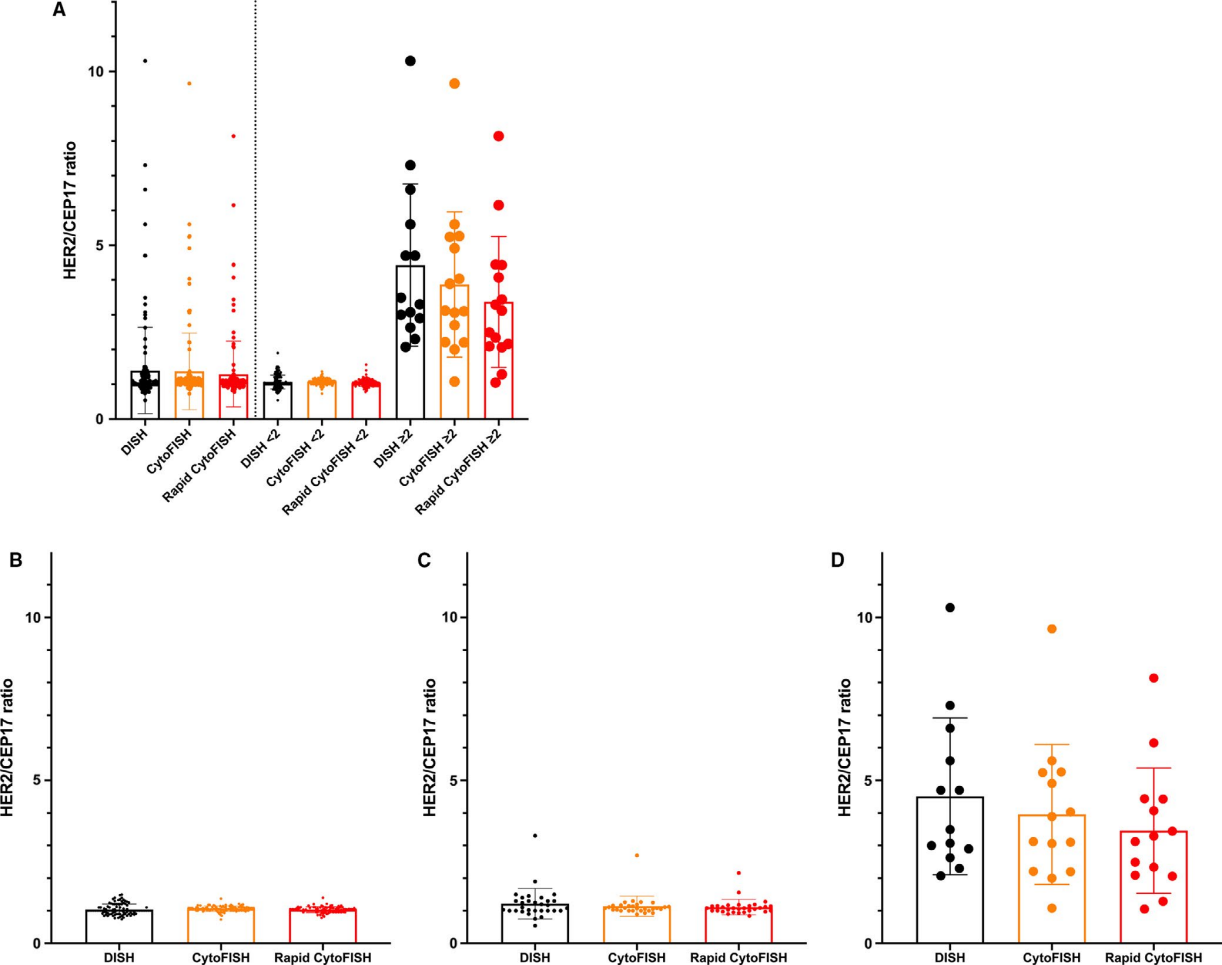
Comparison of HER2/CEP17 ratios between standard FFPE‐tissue DISH and the two CytoFISH protocols. (A) HER2/CEP17 ratios in all samples, and if dividing group by the ratio <2 or ≥2, (B) HER2/CEP17 ratio in HER2‐immunohistochemistry (IHC) 0/1+ samples, (C) in HER2‐IHC 2+ samples, and (D) in HER2‐IHC 3+ samples. There were no significant differences between the standard FFPE‐tissue DISH and the new CytoFISH protocols using cytologic samples (*p* > 0.05, Dunnett's multiple comparison test)

**FIGURE 3:**
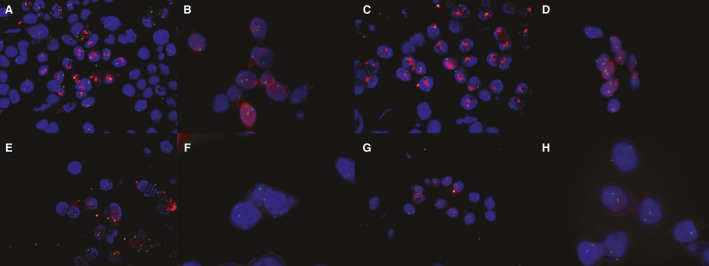
Detection of HER2 on cytology using the new CytoFISH protocols. (A) Gene amplification on imprinted cytology using CytoFISH. (B) Gene amplification on liquid‐based cytology (LBC) using CytoFISH. (C) Gene amplification on imprinted cytology using rapid‐CytoFISH. (D) Gene amplification on LBC using rapid‐CytoFISH. (E) Non‐amplification on imprinted cytology using CytoFISH. (F) Non‐amplification on LBC using CytoFISH. (G) Non‐amplification on imprinted cytology using rapid‐CytoFISH. (H) Non‐amplification on LBC using rapid‐CytoFISH. CEP17 was labeled with Spectrum Green, and HER2 was labeled with Spectrum Orange (Magnification: 100×)

The HER2/CEP17 ratios did not significantly differ between standard FFPE‐tissue DISH and the two CytoFISH protocols (Figure 2, *p* > 0.05). Likewise, the HER2/CEP17 ratios did not significantly differ if dividing group by the ratio <2 or ≥2, if extracting the ductal carcinoma in situ (DCIS) group, if dividing groups according to HER2‐IHC status 0/1+, 2+, or 3+, respectively. Representative examples of FISH on cytologic specimens are shown in Figure 3. HER2/CEP17 signals were observed in 142 specimens (99.3%) when FFPE‐tissue DISH was used for staining, in 139 specimens (97.2%) when CytoFISH was used, and in 139 specimens (97.2%) when rapid‐CytoFISH with AC mixing was used. In addition, polysomy in CEP17 was detected in 10.5% (10 of 95 cases) of HER2 0/1+ patients, 23.3% (7 of 30 cases) in of HER2 2+ patients, and 50% (7 of 14 cases) of HER2 3+ patients. Two HER2‐IHC 2+ cases showed HER2‐ISH diagnostic mismatching between the conventional FFPE‐tissue DISH and CytoFISH because of CEP17 polysomy.

Finally, we divided each of the 143 samples into three parts and performed standard FFPE‐tissue DISH, CytoFISH, and rapid‐CytoFISH with AC mixing. Table 4 shows the HER2 status of each sample based on the HER2/CEP17 ratio and the average HER2 copy number. We found 95.1% agreement between FFPE‐tissue DISH and the two CytoFISH protocols (Cohen's kappa coefficient = 0.771, and 95% confidence interval (CI): 0.614–0.927). When using imprinted cytology, we found 92.0% agreement between FFPE‐tissue DISH and the CytoFISH protocols (Cohen's kappa coefficient = 0.633 and 95% CI: 0.312–0.955). When using LBC, we found 96.8% agreement between FFPE‐tissue DISH and the CytoFISH protocols (Cohen's kappa coefficient = 0.847 and 95% CI: 0.687–1.000). Thus, diagnoses obtained using the new CytoFISH protocols, with and without AC mixing, were nearly equal to those obtained with standard FFPE‐tissue DISH. In just one discrepancy case (HER2‐IHC 3+) between FFPE‐tissue DISH and CytoFISHs, HER2 amplification was seen only in invasive carcinoma in surgical FFPE specimen. This case diagnosed as invasive ductal carcinoma, HER2‐IHC 1+ by preoperative CNB, and demonstrated invasive ductal carcinoma with intratumoral heterogeneity.

**TABLE 4:** HER2 results from standard FFPE‐tissue DISH, CytoFISH, and Rapid‐CytoFISH in all 143 cytology samples

HER2‐IHC	Case	FFPE‐tissue DISH		CytoFISH		Rapid‐CytoFISH	
0	49	Negative	49	Negative	48	Negative	48
		Positive	0	Positive	0	Positive	0
		Not evaluable	0	Not evaluable	1	Not evaluable	1
1+	49	Negative	49	Negative	47	Negative	47
		Positive	0	Positive	0	Positive	0
		Not evaluable	0	Not evaluable	2	Not evaluable	2
2+	31	Negative	30	Negative	28	Negative	28
		Positive	1	Positive	2	Positive	2
		Not evaluable	0	Not evaluable	1	Not evaluable	1
3+	14	Negative	0	Negative	1	Negative	1
		Positive	13	Positive	13	Positive	13
		Not evaluable	1	Not evaluable	0	Not evaluable	0
Kappa value		Standard		0.771		0.771	

Abbreviation: FFPE; formalin‐fixed paraffin‐embedded.

## 4 DISCUSSION

In the present study, we demonstrated that for cytologic breast cancer samples, rapid‐CytoFISH with AC mixing can be used to detect HER2 amplification within the same day, enabling clinicians get both a cancer diagnosis and the HER2 result within 1 day. In addition, although rapid‐CytoFISH provides a result more promptly (within 4 h) than standard FFPE‐tissue DISH, specimens subjected to CytoFISH were very well stained due to the uniform distribution of the cells, superior nuclear chromatin morphology, and smaller amounts of cellular overlap and background debris. This makes the evaluation of HER2 gene amplification using CytoFISH potentially more accurate than with standard ISH methods.

There are two main rapid diagnostic techniques that are used to quickly diagnose breast cancer in any hospital: imprint/smeared cytology and frozen sections, both of which generally entail HE staining. Frozen sections are able to preserve the immune activity of the antigen during processing. They, therefore, exhibit greater reactivity for antigen‐antibody binding than is seen with conventional paraffin sections, and the antigen retrieval step can be omitted. However, in situations where only a small amount of breast tumor is accessible, taking frozen sections may risk depleting the specimen and leaving an inadequate amount of tissue for subsequent IHC or molecular analysis. In contrast, imprint cytology can be done quickly and is more cost effective than frozen sections, and it requires less tissue for production of slides.^25^ Theoretically, our rapid‐IHC and ‐ISH with AC mixing can be applied to both techniques.

At present, CNB represents the gold standard for tumor tissue sampling to evaluate breast lesions. This is because of its low rate inconclusive results and the large amount of histological and molecular information potentially available when clinicians are considering a cancer diagnosis, biomarkers status, or therapeutic options prior to surgery.^26^ CNB has a higher sensitivity (87% vs 74%) and specificity (98% vs 96%) than fine needle aspiration cytology (FNAC), and provides more material for grading tumors and for assessing predictive factors, such as the hormone receptor and HER2 status.^26^, ^27^ Nevertheless, additional cytology, including FNAC and LBC, can be useful because same‐day diagnosis decreases patient anxiety and facilitates prompt treatment. For same‐day diagnoses, cytopathologic diagnosis has advantages in that it is a less expensive, less traumatic, and quicker technique.^27^ Alternatively, core wash cytology specimens can be prepared by washing the tissue contained in the CNB needle notch with saline or cell‐preserving solution, and a preliminary diagnosis can be given to the patient. This is what was done in the present study as we attempted to investigate HER2 status. In addition, the cytopathologic diagnosis from serous effusion in the body cavity is the gold standard for determining whether or not there is cancer metastasis. We will need to determine the usefulness of cytologic specimens for HER2 FISH testing, especially for patients with tumors from which samples are difficult to obtain.

CEP17 abnormalities, including whole chromosome and gene copy number anomalies, allelic losses, and structural rearrangements, are common in breast cancer. CEP17 polysomy is reportedly present in up to 68% of breast cancers.^28^, ^29^ Cytologic specimens are applicable for estimating CEP17 polysomy. The distance between the CEP17 signal and the nearest nuclear membrane is significantly shorter than between the HER2 signal and the nuclear membrane. Because CEP17 is adjacent to the nuclear membrane, it may be lost during sectioning. Consequently, cytologic specimens tend to yield lower HER2/CEP17 ratios, though they are more likely to indicate the “actual” copy numbers.^15^ The CEP17 gain is not due to isolated polysomy, but may instead be gains in the whole chromosome and strongly correlate with aneuploidy with gain of multiple chromosomes.^30^ Although aneuploidy is associated with poor clinical outcomes, irrespective of tumor grade, problems related to aneuploidy and genomic heterogeneity are common among all high‐throughput molecular profiling techniques, including ISH. The response to HER2‐targeted therapy in patients with CEP17 abnormalities is controversial,^31^, ^32^ and accurate assessment of the HER2/CEP17 signal ratio may be crucial for accurate determination of the prognosis of breast cancer patients in clinical practice. For that purpose, HER2 FISH testing of cytologic specimens, which evaluate whole cells may be more accurate than histologic specimens.

Current ASCO/CAP guidelines ^24^ do not provide guidance regarding sample handling, fixation techniques, scoring, or interpretation for use with whole cell cytologic preparations. The 2018 ASCO/CAP guideline focused update recommended the same optimal tissue handling requirements as before, and the only recommendation in cytology is that cytology specimens must be fixed in formalin. Since tissue handling and fixation protocols are different for standard FFPE tissue sections and whole cell cytologic preparations, optimal methods will need to be established for sample handling for FISH. While it is true that HER2 and CEP 17 copy numbers may be more accurate in whole nuclei than in the truncated nuclei present in standard FFPE tissue sections, further investigation will be needed to evaluate the optimal methods for cytologic sample handling.

Rapid‐CytoFISH has several potential limitations. First, the disadvantage of cytologic ISH involves distinguishing invasive areas from heterogeneity. Although there was just one such case in which HER2 results differed between the FFPE‐tissue DISH and CytoFISH in this study, this disadvantage highlights this limitation. That is, there is the potential for selection and possible allocation bias, which are the main pitfall of histological tissue comparison studies. Second, our study has the important potential confounding the effects of including DCIS and clinicians do not know how this might be avoided if imprint cytology of breast tissue is to be used for the determination of HER2 status. The important third limitation of this study is that there is very little information currently available from clinical trials to inform the interpretation of HER2 FISH on whole cells and no guidelines on specimen processing, assay performance, interpretation, and cutoffs. Additional data, new assay performance criteria and cutoff data prior to any clinical use of this methodology in whole cells from breast tissue will be needed. Thus, future research is needed to provide the additional data required to complete this new diagnosing system.

In conclusion, we have shown that CytoFISH could potentially serve as a clinical tool for prompt determination of HER2 status in breast cancer cytology. HER2 rapid‐CytoFISH preserves both the cellular architecture and nuclear morphology, and does not deplete the specimen available for subsequent molecular analysis. This technique facilitates rapid hybridization by taking advantage of the noncontact mixing effect of an applied AC electric field, which enables breast cancer diagnoses and the results of HER2 tests to be obtained on the same day the patient comes to a clinic or hospital.

## CONFLICT OF INTEREST

The authors made no disclosures.

## Data Availability

Data are available upon reasonable request.
